# Curcumin and its nano-formulations: Defining triple-negative breast cancer targets through network pharmacology, molecular docking, and experimental verification

**DOI:** 10.3389/fphar.2022.920514

**Published:** 2022-08-08

**Authors:** Zhicheng Deng, Guanghui Chen, Yonghui Shi, Ying Lin, Jiebin Ou, Hua Zhu, Junyan Wu, Guocheng Li, Li Lv

**Affiliations:** ^1^ Department of Pharmacy, Sun Yat-Sen Memorial Hospital, Sun Yat-Sen University, Guangzhou, China; ^2^ Shenshan Central Hospital, Sun Yat-Sen Memorial Hospital, Sun Yat-Sen University, Shanwei, China; ^3^ Department of Pharmacy, Zengcheng District People’s Hospital of Guangzhou, The Fourth Affiliated Hospital of Guangzhou Medical University, Guangzhou, China; ^4^ Guangdong Provincial Key Laboratory of Malignant Tumor Epigenetics and Gene Regulation, Sun Yat-Sen Memorial Hospital, Sun Yat-Sen University, Guangzhou, China

**Keywords:** curcumin, triple-negative breast cancer, network pharmacology, JAK-STAT signaling pathway, nanoparticles

## Abstract

**Background:** Curcumin (CUR) displays the capability of suppressing the proliferation and metastasis of various cancer cells. However, the effects and underline mechanisms of CUR to treat triple-negative breast cancer (TNBC) have not been systematically elucidated with an appropriate method.

**Methods:** In the present research, a combination method of network pharmacology, molecular docking, and *in vitro* bio-experiment was used to investigate the pharmacological actions and underline mechanisms of CUR against TNBC. First, common targets of CUR and TNBC were screened *via* Venny 2.1.0 after potential CUR-related targets and targets of TNBC were got from several public databases. Then, the Gene Ontology (GO) function and the Kyoto Encyclopedia of Genes and Genomes (KEGG) pathway enrichment were performed on the Metascape website, and the network of compound-targets-pathways was constructed *via* Cytoscape software. Moreover, the network of protein-protein interaction was constructed by the STRING database to screen potential targets. Moreover, molecular docking was applied to affirm the interaction of CUR with the screened top 10 potential targets. Finally, *in vitro* experiments were used to further verify the effects and mechanisms of CUR and its nano-formulation (CUR-NPs) against TNBC.

**Results:** Forty potential targets of CUR against TNBC were obtained. STAT3, AKT1, TNF, PTGS2, MMP9, EGFR, PPARG, NFE2L2, EP300, and GSK3B were identified as the top 10 targets of CUR against TNBC. *In vitro* experiment verified that CUR and CUR-NPs could not only restrain the invasion, migration, and proliferation of MDA-MB-231 cells but also induce their apoptosis. In addition, molecular docking demonstrated that CUR could bind spontaneously with the screened top 10 targeted proteins, and a real-time PCR experiment demonstrated that both CUR and CUR-NPs could downregulate the genetic expression levels of the 10 targets. Moreover, according to the CUR-targets-pathways network, PI3K-Akt, EGFR tyrosine kinase inhibitor resistance, JAK-STAT, Foxo, and HIF-1 signaling pathways were identified as the important pathways of CUR effects on TNBC. Among them, the inhibiting effects of CUR and CUR-NPs on the JAK-STAT signaling pathway were further verified by the western blot analysis.

**Conclusion:** Taken together, the present research demonstrates that CUR and CUR-NPs have pharmacological effects against TNBC *via* a multi-target and multi-pathway manner.

## Introduction

In recent years, tremendous therapeutic progress has been achieved in the treatment of non-triple-negative breast cancer (non-TNBC), but the therapeutic strategies of TNBC are still limited to conventional chemotherapeutic drugs such as paclitaxel, doxorubicin, and platinum-based agents, which have serious cytotoxicities and side effects (Song et al., 2013; Zhang et al., 2020). So, it is urgent to exploit effective treatments with diminished toxicity for TNBC. Curcumin (CUR), an active ingredient of turmeric, displays the capability of inhibiting the proliferation, invasion, and metastasis of various cancer cells. Borges et al. (2020) found that CUR has the potential capacity to inhibit the growth and progression of head and neck cancer cells. Xu et al. (2014) reported that CUR has the ability to suppress the invasion and migration of FIC133 cells (a human thyroid cancer cell line). However, the effects and underline mechanisms of CUR against TNBC have not been systematically elucidated by appropriate methods.

Network pharmacology is a powerful tool for systematically exploring the complex pharmacological effects of drugs (Han et al., 2021). By the method of network pharmacology, some traditional Chinese medicines have been reported to suppress cancers. Zhang et al. (2021) reported that oxyepiberberine inhibited the development and progress of non-small cell lung cancer (NSCLC) using the combination of network pharmacology and biological experiment. Yuan et al. (2021) found that scopoletin against NSCLC through the pathways of RAS-RAF-MEK-ERK and PI3K/AKT *via* the combination of network pharmacology and molecular docking. So, we hypothesized that the combination of network pharmacology, molecular docking, and *in vitro* bio-experiment will be a powerful tool to systematically investigate the pharmacological actions of CUR against TNBC.

**FIGURE 1:**
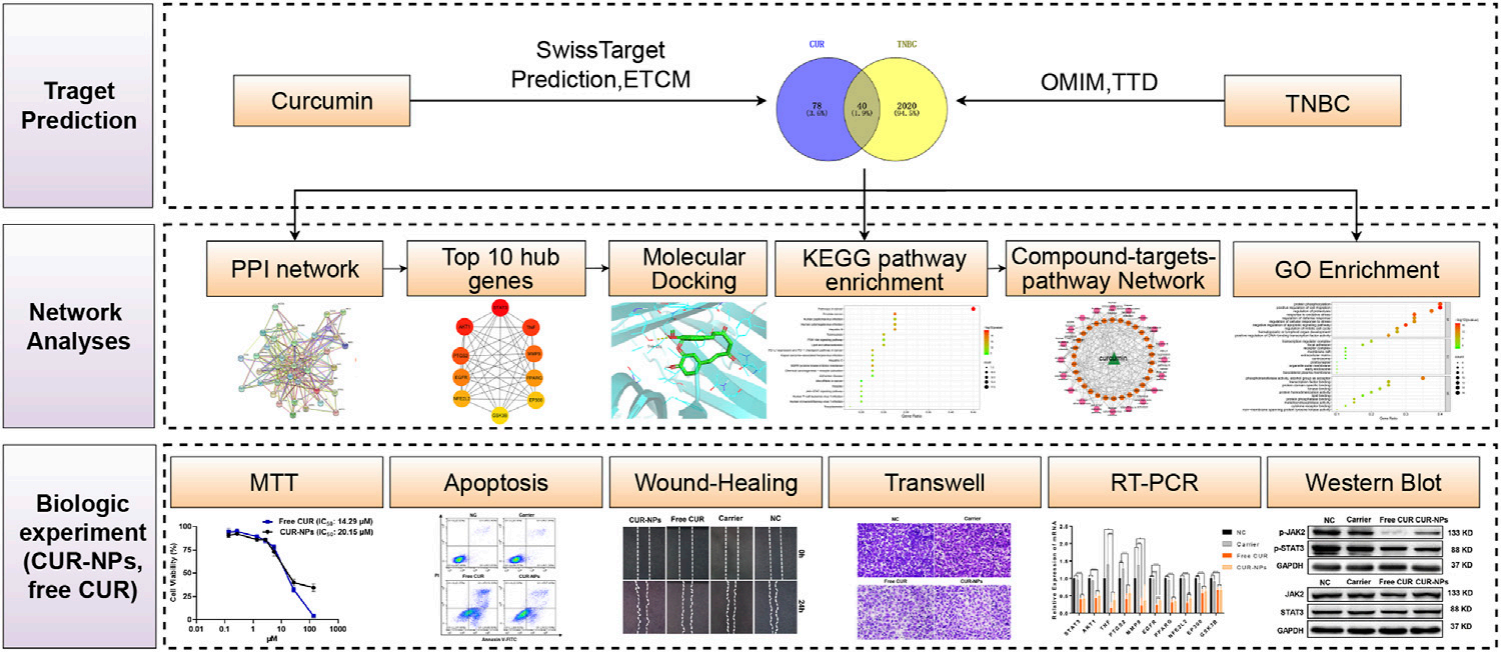
The workflow of this research.

Herein, common targets of CUR and TNBC were screened *via* Venny 2.1.0 after potential CUR-related targets and targets of TNBC were obtained from public databases. Subsequently, the Gene Ontology (GO) function and the Kyoto Encyclopedia of Genes and Genomes (KEGG) pathway enrichment were performed on the Metascape website. The network of compound-targets-pathways was also constructed by Cytoscape software. In addition, the database of Search Tool for the Retrieval of Interacting Genes/Proteins (STRING) was used to construct the network of the protein-protein interaction (PPI) for screening potential targets. Moreover, molecular docking was applied to affirm the interaction of CUR with the screened top 10 potential targets. Finally, *in vitro* bio-experiments were used to further verify the effects and mechanisms of free CUR and CUR-loaded polyethylene glycol_5k_-bock-poly (l-lactide)_5k_ nanoparticles (CUR-NPs) which were prepared by encapsulating CUR with the carrier of polyethylene glycol_5k_-bock-poly (l-lactide)_5k_, (PEG_5k_-b-PPLA_5k_). The workflow of this study is exhibited in Figure 1.

## Materials and methods

### Materials

CUR was purchased from Shanghai D&B biological science and technology Co., Ltd. (Shanghai, China). PEG_5k_-b-PPLA_5k_ was purchased from Jinan Daigang Biomaterial Co., Ltd. (Jinan, China). MDA-MB-231 cells were obtained from the ATCC (Manassas, VA, USA). MTT was supplied by Sigma (St. Louis, MO, USA). Rabbit anti-human anti-STAT3 (BM4052), anti-phospho-STAT3 (Tyr705) (BM4835), anti-JAK2 (BM4165), and anti-phospho-JAK2 (Tyr1007/Tyr1008) (BM4839) antibody products were obtained from Boster Biological Technology Co., Ltd. (Wuhan, China). HRP-labeled Goat Anti-Rabbit IgG (A0208) and the Annexin V-FITC apoptosis detection kit (C1062M) were purchased from Beyotime Biotechnology (Shanghai, China). 24-well transwell chambers (Cat. No. 3422) and polymerized Matrigel (Cat. No. 356234) were bought from Corning (Corning, NY, USA).

### Prediction of potential CUR-related targets

**FIGURE 2:**
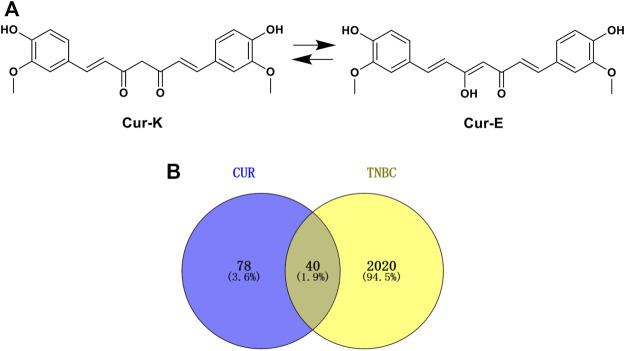
**(A)** 2D structure of CUR. **(B)** The Venn diagram of the common genes of CUR and TNBC.

Potential CUR-related targets were obtained from the Swiss Target Prediction database (Daina et al., 2019) (http://www.swisstargetprediction.ch/) and the Encyclopedia of Traditional Chinese Medicine (ETCM) database (Xu et al., 2019) (http://www.tcmip.cn/ETCM/index.php/Home/). In the Swiss Target Prediction database, potential CUR-related targets were retrieved by the structure of CUR (both keto and enol forms) in Figure 2, and species were limited to “*Homo sapiens*”. In the ETCM database, potential CUR-related targets were searched directly using the keyword of “CUR”. All targets obtained in both the Swiss Target Prediction database and ETCM were selected as potential CUR-related targets.

### Potential targets of CUR against TNBC

First, TNBC-related targets were identified in the Online Mendelian Inheritance in Man database (OMIM, https://omim.org/), the Therapeutic Target Database (Li et al., 2018) (TTD, http://db.idrblab.net/ttd/), and the DisGeNET database (Piñero et al., 2017) (https://www.disgenet.org/) using keywords of “triple negative breast cancer/carcinoma”. Subsequently, Venny 2.1.0 (https://bioinfogp.cnb.csic.es/tools/venny/index.html) was applied to screen the common targets of CUR and TNBC which were defined as the potential targets of CUR against TNBC in the present research.

### GO and KEGG pathway enrichment analysis

GO and KEGG pathway enrichment of the common targets of CUR and TNBC were analyzed on the Metascape website (https://metascape.org/). Molecular function (MF), cell component (CC), and biological process (BP) were included in the GO analysis. The analyses were carried out using a *p* value of less than 0.01. The top 10 items of GO and the top 20 items of the KEGG pathway were selected and visualized on the Bioinformatics website (http://www.bioinformatics.com.cn/).

### The network construction of compound-targets-pathways

The software of Cytoscape 3.7.1 (https://cytoscape.org/download.html) was applied to construct and analyze the network of CUR, targets, and pathways based on KEGG pathway enrichment analysis for understanding the complex relationships among them.

### Construction of the PPI network

The PPI network of potential targets of CUR against TNBC was constructed by the database of STRING (Szklarczyk et al., 2019) (https://string-db.org/), using common targets of CUR and TNBC with minimum required interaction score ≥0.4, and species limited to “*Homo sapiens*”. After download, the TSV format of the PPI network was imported into the Cystoscope software to screen the top 10 targets of CUR against TNBC by using the maximal clique centrality (MCC) algorithm of the CytoHuba plug-in.

### Molecular docking

The structure of CUR and the top 10 targets obtained from PPI network analysis were downloaded from the website of PubChem and the database of RSCB PDB (https://www.rcsb.org/), respectively. Autodock Vina (v1.1.2.0) was used for molecular docking in our study (Trott and Olson, 2010), and the binding pocket of CUR with the potential protein target was defined by AutoDockTools (v1.5.7) based on the binding site of the co-crystallized ligand in the crystal structure of the complex (Morris et al., 2009; Zhang et al., 2019). After separating from the corresponding complex by AutoDockTools, the co-crystallized ligand was re-docked and scored (Shi et al., 2015). Finally, the PyMOL software was applied to visualize the optimum conformations of CUR and its targets.

### Preparation of CUR-NPs

CUR-NPs were prepared by the thin-film hydration method with some modifications (Emami et al., 2018). In brief, 50 mg of PEG_5k_-b-PLLA_5k_, 5 mg of CUR, and 20 ml of dichloromethane were added into a 100 ml round bottom single neck flask. After complete dissolution, the dichloromethane was removed by a rotary evaporator at 40°C with a rotation rate of 100 rpm to form a thin film. 10 ml of ultrapure water was added to dissolve the formed thin-film. Then, the solution was centrifugated with a speed of 4,000 rpm for 10 min. At the end of centrifugation, the nanoparticle solution in the supernatant was collected for further study.

### Characterization of nanoparticles

The size and zeta potential of the prepared CUR-NPs were measured by dynamic light scattering (DLS) with a Malvern zetasizer (MALVERN zetasizer nano ZEN3700). The morphology of the prepared CUR-NPs was observed by a transmission electron microscope (JEOL JEM-2100).

### Quantification of CUR

A standard calibration of CUR (0.27, 2.71, 13.57, 54.29, 135.73 μM) was prepared with dimethylsulfoxide (DMSO). Then, the CUR concentration of CUR-NPs after dilution with DMSO was quantified using the prepared standard calibration of CUR by a SPARK 10M plate reader (TECAN, Switzerland) at 470 nm.

### Drug release

The CUR release from the prepared CUR-NPs *in vitro* was studied in PBS (pH7.4). Briefly, 1 ml of the prepared CUR-NPs solution was added to a dialysis bag (MWCO = 3,500 Da). Then, the dialysis bag containing CUR-NPs solution was immersed in 30 ml of PBS (pH7.4) with 0.2% tween-80 at 37°C. The external buffer solution was substituted with fresh PBS (pH7.4) with 0.2% tween-80 at predetermined time intervals. Concentrations of CUR in the external butter solution were measured using the method described in the “Quantification of CUR”.

### MTT assay

The cytotoxic effects of free CUR, CUR-NPs, and PEG_5k_-b-PLLA_5k_ on MDA-MB-231 cells were determined by the MTT assay. MDA-MB-231 cells were seeded into 96-well plates at a density of 3 × 10^3^ cells/well and cultured overnight. Afterward, free CUR (in DMSO) or CUR-NPs was added into each well with concentrations of CUR from CUR from 0.027 to 271.46 µM for 48 h; PEG_5k_-b-PLLA_5k_ was added into each well with concentrations from 0.1 to 100 µM for 48 h. Thereafter, the culture media were replaced with 10 µL of the MTT solution (the concentration of the MTT solution was 12 mM) after washing with PBS three times and incubated for another 4 h. At the end of incubation, 100 µL of DMSO was added into each well after the culture medium was discarded. The optical density was measured by the Spark M10 multimode plate reader (Tecan, Männedorf, Switzerland) at 570 nm. The half-inhibitory concentrations (IC_50_) of different groups were calculated using the software GraphPad Prism 7.

### Apoptosis assay

The apoptotic rates of MDA-MB-231 cells after different treatments were detected using the method of Annexin V-FITC/PI double staining. Briefly, MDA-MB-231 cells were seeded into 6-well plates (1 × 10^5^ cells/well) and cultured overnight. Afterward, free CUR, CUR-NPs, or PEG_5k_-b-PLLA_5k_ was added into each well with a CUR concentration of 27.15 µM or PEG_5k_-b-PLLA_5k_ concentration of 10 µM for 48 h. Then, cells were harvested after washing with PBS three times. 5 μL of Annexin V-FITC and 10 μL of PI were added to the harvested cells after the cells were resuspended in 195 μL 1×binding buffer. Flow cytometric analyses were performed on a CytoFLEX (Beckman, USA) utilizing 10,000 events after the cells were incubated for another 10–20 min.

### Wound-healing assay

MDA-MB-231 cells were seeded into 6-well plates (5 × 10^5^ cells/well) with 10% FBS until they reached confluence, and a 10 μL pipette tip was used to create wounds. Free CUR, CUR-NPs, or PEG_5k_-b-PLLA_5k_ was added into each well with a CUR concentration of 13.57 µM or a PEG_5k_-b-PLLA_5k_ concentration of 5 µM. The images of migration were captured with a ZEISS Observer A1 inverted microscope (×10 objective) (Carl Zeiss, Oberkochen, Germany).

### Transwell assay

Transwell chambers in 24-well plates were coated with 100 μL of polymerized matrigel (30 μg), followed by seeding of MDA-MB-231 cells at a density of 1 × 10^5^ cells/200 µL serum-free culture medium. Free CUR, CUR-NPs, or PEG_5k_-b-PLLA_5k_ were added into each well with a CUR concentration of 13.57 µM or a PEG_5k_-b-PLLA_5k_ concentration of 5 μM, and 600 µL culture media containing 10% FBS were adding to the lower chamber. After 12 h incubation at 37°C, the cells on the lower surface were obtained by staining with a 0.1% crystal violet solution. The numbers of transmembrane cells were counted after three images of five random fields of each membrane were captured using a Nikon Ni-U microscope (Tokyo, Japan).

### Relative quantification by real-time PCR

MDA-MB-231 cells were seeded into 6-well plates (1 × 10^5^ cells/well), and then free CUR, CUR-NPs, or PEG_5k_-b-PLLA_5k_ were added into each well with a CUR concentration of 13.57 µM or a PEG_5k_-b-PLLA_5k_ concentration of 5 µM for 48 h. Afterward, total RNA was isolated from MDA-MB-231 cells with the TRIzol^®^ reagent, and cDNA synthesis was conducted with the RevertAid First Stand cDNA Synthesis Kit. Real-time PCR (RT-PCR) was then conducted using Hieff^®^ qPCR SYBR^®^ Green Master Mix (Yeasen Biotechology). Primers used for RT-PCR were *STAT3* (Forward 5′-GCC​AAT​TGT​GAT​GCT​TCC​CT-3′; Reverse 5′- TCT​TGG​GAT​TGT​TGG​TCA​GC-3′), *AKT1* (Forward 5′-GCT​GCA​CAA​ACG​AGG​GGA​G-3′; Reverse 5′-CCT​CAC​GTT​GGT​CCA​CAT​CC-3′), *TNF* (Forward 5′-CTG​CAC​TTT​GGA​GTG​ATC​GG-3′; Reverse 5′-TCA​GCT​TGA​GGG​TTT​GCT​AC-3′), *PTGS2* (Forward 5′-GAA​AAC​TGC​TCA​ACA​CCG​GAA-3′; Reverse 5′-TTG​CAT​TTC​GAA​GGA​AGG​GA-3′), *MMP9* (Forward 5′-TTC​CAG​TAC​CGA​GAG​AAA​GCC-3′; Reverse 5′-CAT​AGG​TCA​CGT​AGC​CCA​CT-3′), *EGFR* (Forward 5′-GAG​AAC​TGC​CAG​AAA​CTG​ACC-3′; Reverse 5′-GTG​GCT​TCG​TCT​CGG​AAT​TTG-3′), *PPARG* (Forward 5′-GGG​GAT​GTC​TCA​TAA​TGC​CAT​CAG-3′; Reverse 5′-TTG​CTT​TGG​TCA​GCG​GGA​AG-3′), *NFE2L2* (Forward 5′-GTT​GCC​CAC​ATT​CCC​AAA​TCA-3′; Reverse 5′-ACG​TAG​CCG​AAG​AAA​CCT​CAT-3′), *EP300* (Forward 5′-CAG​CGA​TGG​CAC​AGA​TTT​TGG-3′; Reverse 5′-TAG​GGG​AAC​TAC​CAG​ATC​GCA-3′), GSK3B (Forward 5′-TCC​AGT​GGT​GAG​AAG​AAA​GAT​GA-3′; Reverse 5′-GCG​TCT​GTT​TGG​CTC​GAC​TA-3′), *β-ACTIN* (Forward 5′-CCA​ACC​GCG​AGA​AGA​TGA-3′; and Reverse 5′-CCA​GAG​GCG​TAC​AGG​GAT​AG-3′).

### Western blot

MDA-MB-231 cells were seeded into 6-well plates (1 × 10^5^ cells/well), and then free CUR, CUR-NPs, or PEG_5k_-b-PLLA_5k_ were added into each well with a CUR concentration of13.57 µM or a PEG_5k_-b-PLLA_5k_ concentration of 5 µM for 48 h. Proteins were extracted using a radio-immuno-precipitation assay buffer, and the extracted protein samples were transferred to nitrocellulose membranes after boiling with a 5×loading buffer and separating by 10% SDS-polyacrylamide gel electrophoresis. The membranes were then blocked with 5% skim milk for 1 h at room temperature, followed by incubating with one of the following antibodies at indicated dilution for about 12 h at 4°C: anti-STAT3 (1:1,000), anti-phospho-STAT3 (1:750), anti-JAK2 (1:1,000), anti-phospho-JAK2 (1:750), and anti-GAPDH (1:2,000). Then, the membranes were washed with 1×TBST buffer three or four times, followed by incubating with secondary HRP-conjugated goat anti-Rabbit IgG (1:1,000) for 1 h at room temperature. After washing with TBST buffer, the membranes were visualized using the Ultra-sensitive ECL chemiluminescence kit (Beyotime Biotechnology), and the gray scales of the bands were semi-quantitated by VisionWorks software (UVP, Upland, CA).

### Statistical analysis

The data were analyzed *via* One-way Analysis of Variance (ANOVA) by the IBM SPSS Statistics 23 software (SPSS Inc., Chicago, IL, USA). The difference was considered statistically significant for all tests when *p* < 0.05.

## Results

### Potential targets of CUR against TNBC

Figure 2A exhibited the 2D structure of CUR and potential targets of CUR obtained from the Swiss Target Prediction database and ETCM database (Supplementary Table S1). Among these potential targets, 110 targets were obtained in the Swiss Target Prediction database and eight targets were got in the ETCM database. Based on the OMIM, TTD, and DisGeNET database, 2060 TNBC-related targets were identified after removing duplication (Supplementary Table S2). Then, 40 potential targets of CUR against TNBC were screened by the Venny 2.1.0 (Figure 2B, Supplementary Table S3).

### GO and KEGG enrichment analysis

**FIGURE 3:**
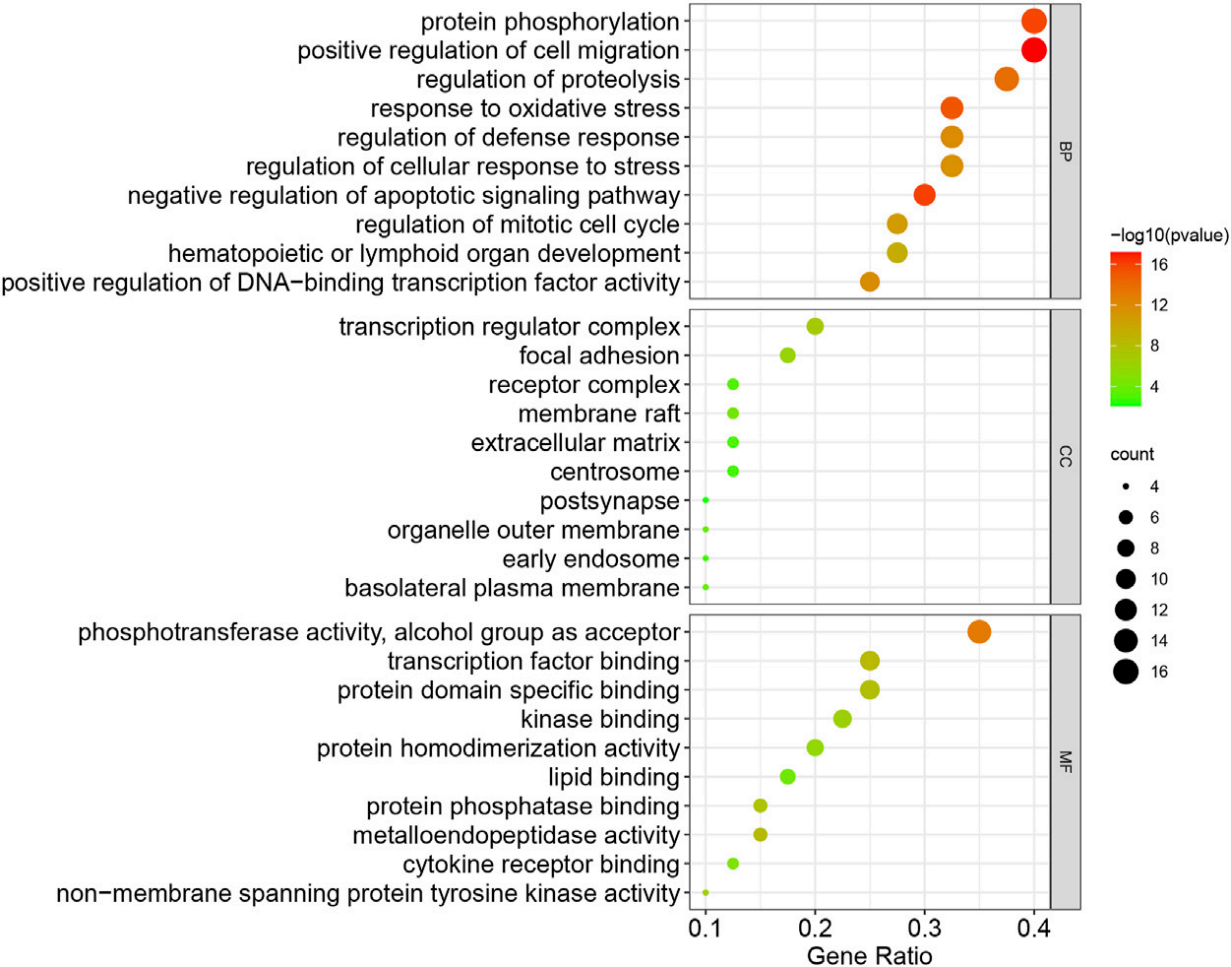
The top 10 of BP, CC, and MF in the GO enrichment analysis of the common targets of CUR and TNBC. (BP, biological process; CC, cellular component; MF, molecular function).

**FIGURE 4:**
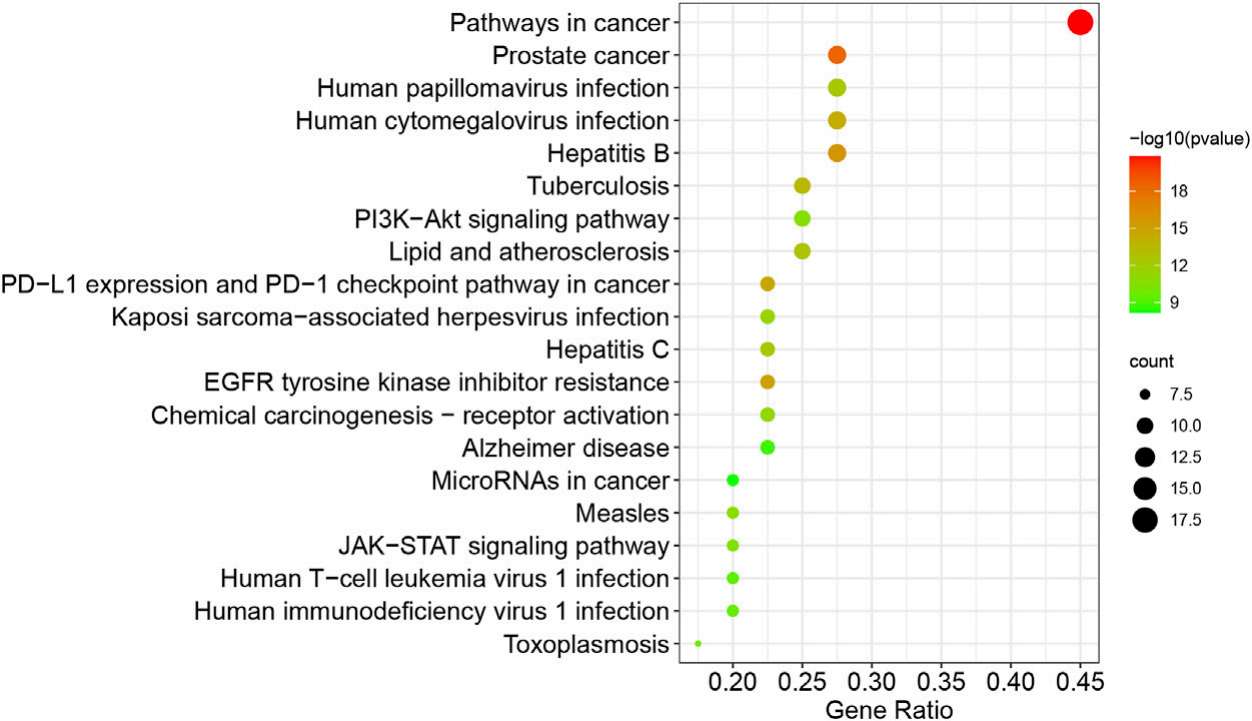
The top 20 pathways of the KEGG enrichment analysis of the common targets of CUR and TNBC.

To illustrate the anti-TNBC mechanisms of CUR, the GO and KEGG pathway enrichment of the 40 common targets of CUR and TNBC were investigated. As shown in Figure 3, the common targets were significantly enriched in positive regulation of cell migration, protein phosphorylation, regulation of proteolysis, response to oxidative stress, regulation of defense response, regulation of cellular response to stress, negative regulation of apoptotic signaling pathway, positive regulation of DNA-binding transcription factor activity, hematopoietic or lymphoid organ development, and regulation of mitotic cell cycle for BP enrichment analysis; transcription regulator complex, focal adhesion, membrane raft, receptor complex, extracellular matrix, centrosome, basolateral plasma membrane, organelle outer membrane, early endosome, and postsynapse for CC enrichment analysis; the phosphotransferase activity alcohol group as acceptor, transcription factor binding, protein domain specific binding, kinase binding, protein homodimerization activity, lipid binding, metalloendopeptidase activity, protein phosphatase binding, cytokine receptor binding, non-membrane spanning protein tyrosine kinase activity for MF enrichment analysis. As for KEGG pathway analysis, the significant pathways associated with breast cancer were EGFR tyrosine kinase inhibitor resistance, the PI3K-Akt signaling pathway, PD-L1 expression and PD-1 checkpoint pathway in cancer, hemical carcinogenesis-receptor activation, JAK-STAT signaling pathway, and MicroRNAs in cancer (Figure 4).

### Compound-targets-pathways network construction and analysis

**FIGURE 5:**
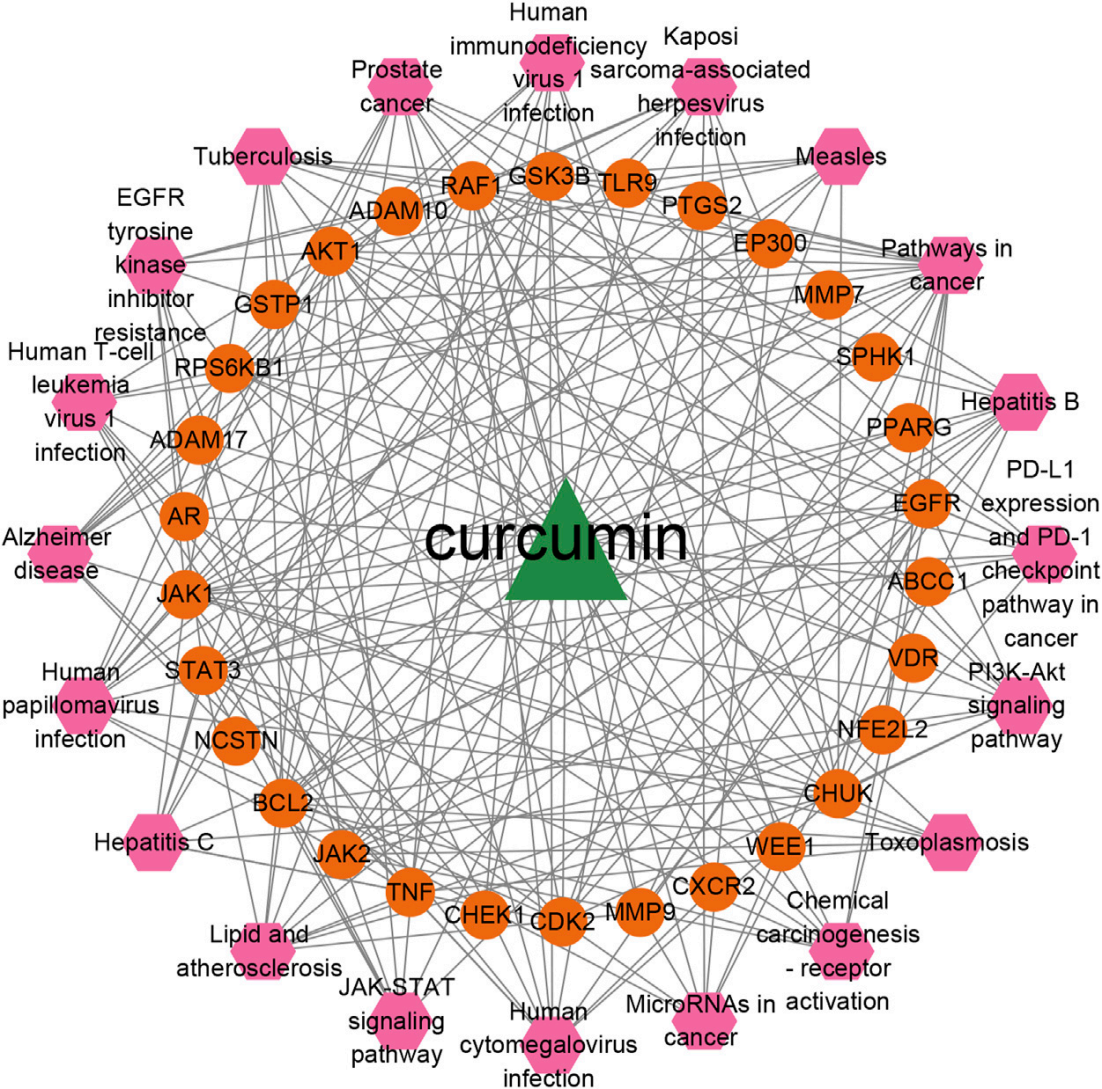
The network of the top 20 pathways and corresponded targets of CUR against TNBC.

Based on the top 20 signaling pathways and the genes enriched in these signaling pathways, a network of CUR-targets-pathways was established. As shown in Figure 5, the constructed network contains 51 nodes (CUR, 30 targets, and 20 pathways) and 223 edges. The triangle, ellipse, and hexagon represent CUR, targets, and pathways, respectively; the edges indicate the relationships among them. It was found that CUR interacted with many targets and pathways, indicating that CUR against TNBC through multiple targets and pathways. The degree values of different pathways were calculated using the plug-in of cytoNCA. Among the top 20 signaling pathways, PI3K-Akt signaling pathway (degree = 10), EGFR tyrosine kinase inhibitor resistance (degree = 9), hemical carcinogenesis-receptor activation (degree = 9), PD-L1 expression and PD-1 checkpoint pathway in cancer (degree = 9), JAK-STAT signaling pathway (degree = 8), and MicroRNAs in cancer (degree = 8) were specific pathways which may play important roles in the effects of CUR against TNBC.

### PPI of the potential targets of CUR against TNBC

**FIGURE 6:**
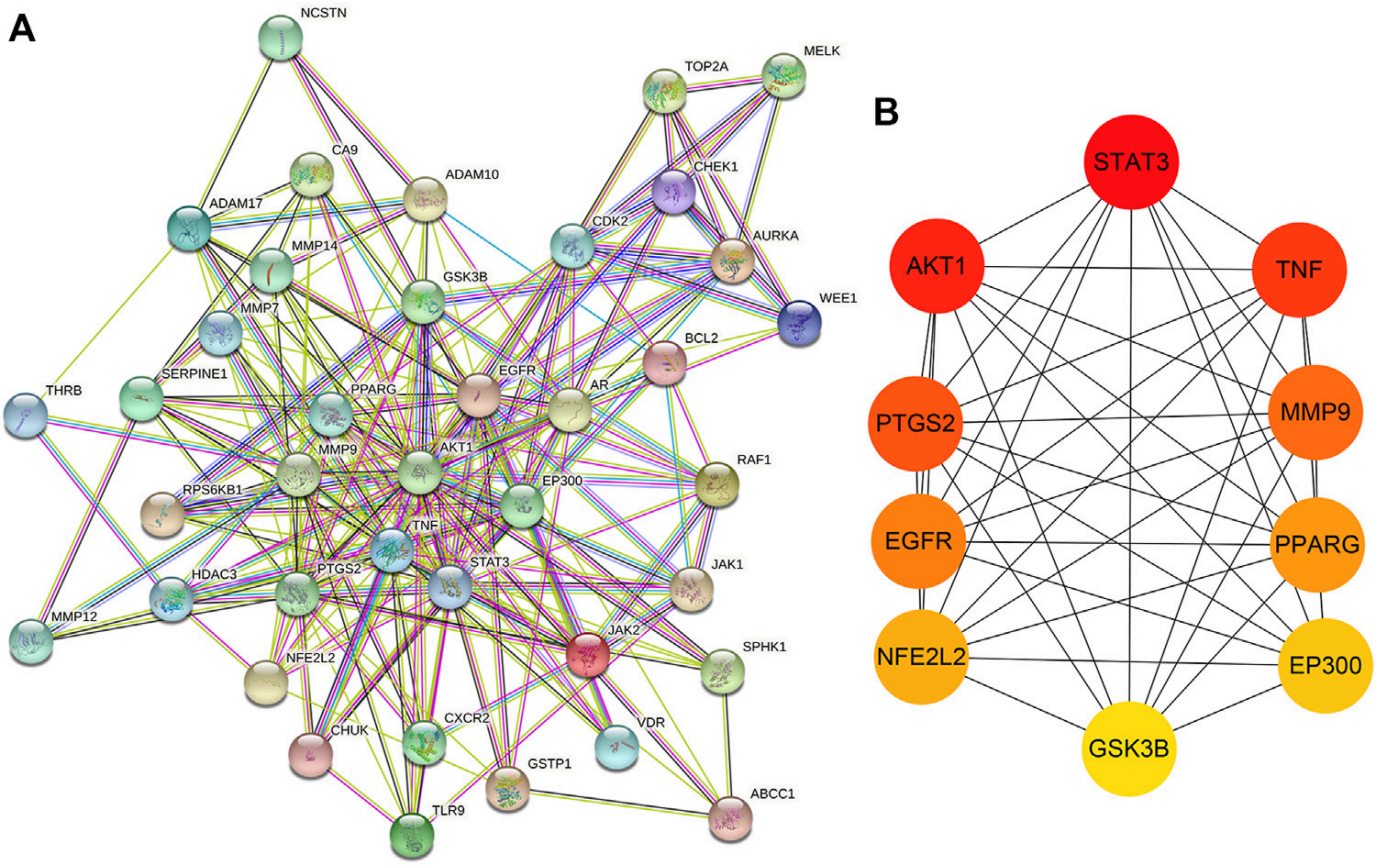
The PPI network and top 10 targets of CUR against TNBC. **(A)** The PPI network of potential targets of CUR against TNBC. **(B)** The interaction diagram of the top 10 targets of CUR against TNBC.

A PPI network was acquired by STRING to illustrate the relationships between the 40 common targets of CUR and TNBC. The PPI network has 40 nodes and 223 edges, and the average node degree of the constructed network is 11.2; the PPI enrichment *p* value is less than 1.0 e^−16^, and the local clustering coefficient is 0.642 (Figure 6A). Subsequently, the constructed network was further investigated to screen the top 10 targets by MCC scores, and results were exhibited in Figure 6B and Table 1. The algorithm of MCC is one of the local-based methods which directly analyze neighborhood of a vertex to cluster featured nodes and it can capture the most essential proteins in the PPI network with both high-degree and low-degree proteins (Chin et al., 2014). The higher the MCC score is, the more essential role the protein plays. According to the MCC scores, the top 10 targets are STAT3, AKT1, TNF, PTGS2, MMP9, EGFR, PPARG, NFE2L2, EP300, and GSK3B, which may play important roles in the constructed PPI network of potential targets of CUR against TNBC.

**TABLE 1:** The scores of the top 10 targets of CUR against TNBC calculated by the MCC method.

Rank	Name	Score
1	STAT3	50,208
2	AKT1	49,208
3	TNF	49,068
4	PTGS2	45,144
5	MMP9	31,944
6	EGFR	30,846
7	PPARG	30,432
8	NFE2L2	21,000
9	EP300	17,186
10	GSK3B	15,193

### Molecular docking and analysis

The top 10 protein targets (STAT3, AKT1**,** TNF, PTGS2, MMP9, EGFR, PPARG, NFE2L2, EP300, and GSK3B) from the results of PPI network analysis were chosen for further validation *via* a method of molecular docking by the Autodock Vina software (v1.1.2.0). CUR creates hydrogen bonds in the blinding pockets of the aforementioned 10 proteins (Supplementary Figures S1–S10). The binding energy was used to score the binding affinity of ligand and receptor. It is generally acknowledged that binding energy<0 kcal/mol indicates the interaction of ligand and receptor is spontaneous, and binding energy < -5 kcal/mol means that the ligand and receptor may assemble (Feng et al., 2021; Ni et al., 2021; Li et al., 2022a; Li et al., 2022b). The docking scores of CUR or the co-crystallized ligand with the selected protein targets (receptors) were shown in Table 2. From these results, it could be found that the binding energies of CUR or the co-crystallized ligand with all the 10 receptors were below -5 kcal/mol, indicating that CUR might bind spontaneously with these receptors like the corresponding co-crystallized ligands. However, the binding abilities of compounds with protein targets in molecular modeling and experiments are not always consistent, due to the limitations of molecular docking. For example, many molecular docking procedures fail to adequately consider the effect of water molecules on hydrogen bonds during ligand binding (Chen et al., 2021). So, experiments were done in our study to further verify these potential targets of CUR against TNBC.

**TABLE 2:** The binding energies of CUR or co-crystallized ligands with related protein targets.

Name	PDB ID	Binding energy (kcal/mol)	
		CUR	Co-crystallized ligands
STAT3	6NJS	−6.3	−9.1
AKT1	4EJN	−9.2	−13.9
TNF	6OOZ	−10.0	−12.5
PTGS2	5F1A	−8.8	−10.9
MMP9	4HMA	−8.2	−13.6
EGFR	1M17	−7.9	−7.4
PPARG	5UGM	−8.5	−10.6
NFE2L2^a^	3ZGC	−7.8	—
EP300	5LKT	−9.7	−8.9
GSK3B	4J71	−7.4	−5.6

a Because the co-crystallized ligand of NFE2L2 in the database of RSCB PDB (https://www.rcsb.org/) is not a small molecular ligand, the co-crystallized ligand was not re-docked and scored.

### Preparation and characterization of CUR-NPs

**FIGURE 7:**
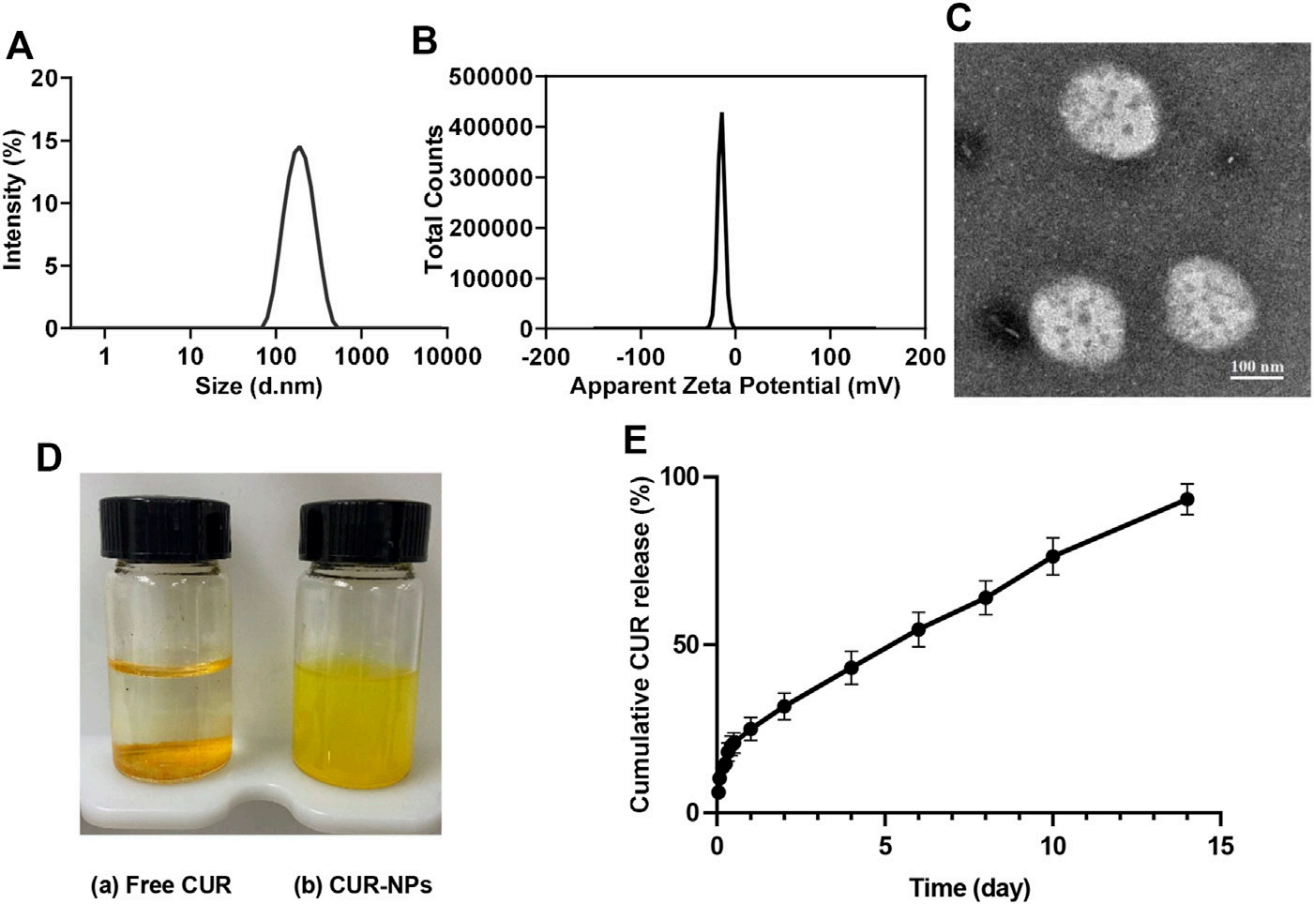
Characterization of CUR-NPs. **(A)** The hydrodynamic diameter of CUR-NPs. **(B)** The zeta potential of CUR-NPs. **(C)** The TME image of CUR-NPs. **(D)** The appearances of free CUR(a) and CUR-NPs(b) dissolved in ultrapure water. **(E)** *In vitro* CUR release from CUR-NPs.

The application of CUR in the clinic is limited by its poor water solubility. So, CUR-NPs were prepared to increase their water solubility. CUR-NPs were prepared with a thin-film hydration method. As depicted in Figures 7A–C **,** the size of CUR-NPs was 120 nm with spherical morphology and zeta potential of -5 mv. CUR-NPs with size in the range of 8–200 nm is thought to have excellent capability of accumulating in the tumor tissue with the enhanced permeability and retention (EPR) effect because the renal clearance threshold is 8 nm and the average gap of leaking blood vessels in cancer tumors is about 200 nm (Peer et al., 2007; Lv et al., 2014). As can be noted from Figure 7D, the solubility of CUR in water is limited ((i.e.≈29.86 nM (Kaminaga et al., 2003)). In contrast, CUR-NPs are a colloidal solution and achieve CUR concentration up to 5,184,863.46 nM, which is about 173,639-fold enhancement compared with free CUR. Moreover, the drug-release behavior was investigated in PBS (pH7.4). The released profile of CUR-NPs displayed a biphasic release pattern, and about 24% of CUR was released from the prepared CUR-NPs in the first 24 h, following that a sustained drug-release behavior was found (Figure 7E). The biphasic release patterns are commonly seen in drug-loaded nanoparticles. Kumari et al. (2016) prepared CUR-loaded mPEG-PLA micelles by a thin-film hydration method, and 30% of CUR was fast released from the micelles within the first 12 h, followed by a sustained drug-release behavior. Gao et al. (2013) prepared CUR-loaded mPEG-PLA micelles by a self-assembly method, about 20% of CUR was released in the initial fast release phase. An ideal anticancer nanoparticle is one that has no drug leakage before reaching the tumor targets. The initial rapid release of drug from the drug-loaded nanoparticle may induce the drug leakage in blood circulation before reaching target sites, which is a common problem of drug-loaded nanoparticles and it is very difficult to solve at present.

### Effects on cell proliferation and apoptosis

**FIGURE 8:**
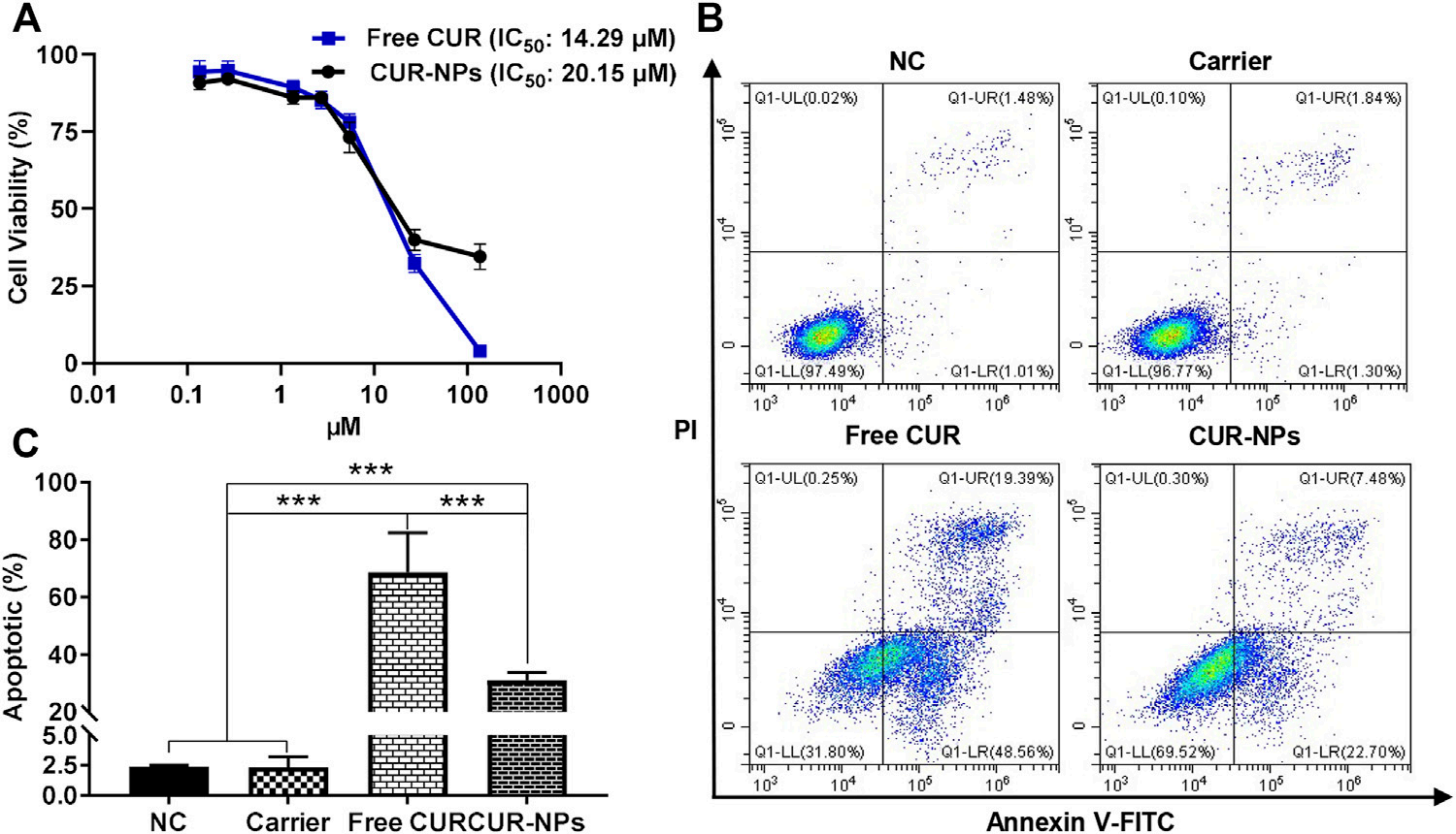
CUR and CUR-NPs inhibit proliferation and induce apoptosis of MDA-MB-231 cells. **(A)** Effects of CUR and CUR-NPs on inhibiting the proliferation of MDA-MB-231 cells. **(B)** The cell apoptosis was investigated by Annexin V-FITC/PI staining. **(C)** Histogram analysis showed the percentage of apoptosis. NC, treated with negative control; carrier, treated with the carrier of PEG_5k_-b-PLLA_5k_; CUR, treated with free CUR; CUR-NPs, treated with CUR-loaded PEG_5k_-b-PLLA_5k_ nanoparticles. ***p < 0.001.

After being treated with gradient doses of CUR in the formation of free CUR or CUR-NPs for 48 h, the viabilities of MDA-MB-231 cells were assayed using the MTT method. As exhibited in Figure 8A, both free CUR and CUR-NPs restrained the viabilities of MDA-MB-231 cells in a dose-dependent manner. The IC_50_ value of free CUR against MDA-MB-231 cells was 14.29 μM which was lower than the treatment of CUR-NPs (20.15 μM). Moreover, the cytotoxicity of the carrier (PEG_5k_-b-PLLA_5k_) against MDA-MB-231 cells was also explored, and the result showed that PEG_5k_-b-PLLA_5k_ has negligible cytotoxicity against MDA-MB-231 cells at the concentration used to prepare CUR-NPs (Supplementary Figure S11). The capabilities of free CUR, CUR-NPs, and the carrier (PEG_5k_-b-PLLA_5k_) to induce the apoptosis of MDA-MB-231 cells were investigated by Annexin V-FITC/PI double staining. As depicted in Figures 8B,C, in the free CUR-treated group and the CUR-NPs-treated group, the apoptotic rates of MDA-MB-231 cells were 68.58% and 31.02%, respectively, both were significantly higher than that of treatment with PEG_5k_-b-PLLA_5k_ or the negative control. These results demonstrated that both free CUR and CUR-NPs have the ability to restrain cell proliferation of MDA-MB-231 cells and induce their apoptosis *in vitro*. Compared with free CUR, the lower anti-proliferation and induced-apoptosis ability of CUR-NPs *in vitro* may be partly attributed to that only 30% of CUR was released from CUR-NPs to have effects on cancer cells after 48 h of incubation. Moreover, there are many other factors that affect the anti-proliferation and induced-apoptosis of CUR such as cellular uptake and metabolism. It was reported that the cellular uptake and metabolism of CUR play an important role in the cytotoxicities of CUR against hormone-dependent ZR-75-1 and hormone-independent MDA-MB-231 breast cancer cells (Jamil et al., 2017). The uptake of nanoparticles by cells is mainly *via* the endocytosis route; however, free drugs are mainly *via* free diffusion or active transport (Lv et al., 2014; Cojocaru et al., 2020). So, the exact reasons for the difference between free CUR and CUR-NPs in the biological effects need to be further investigated in the future.

### Suppression of cell migration and invasion

**FIGURE 9:**
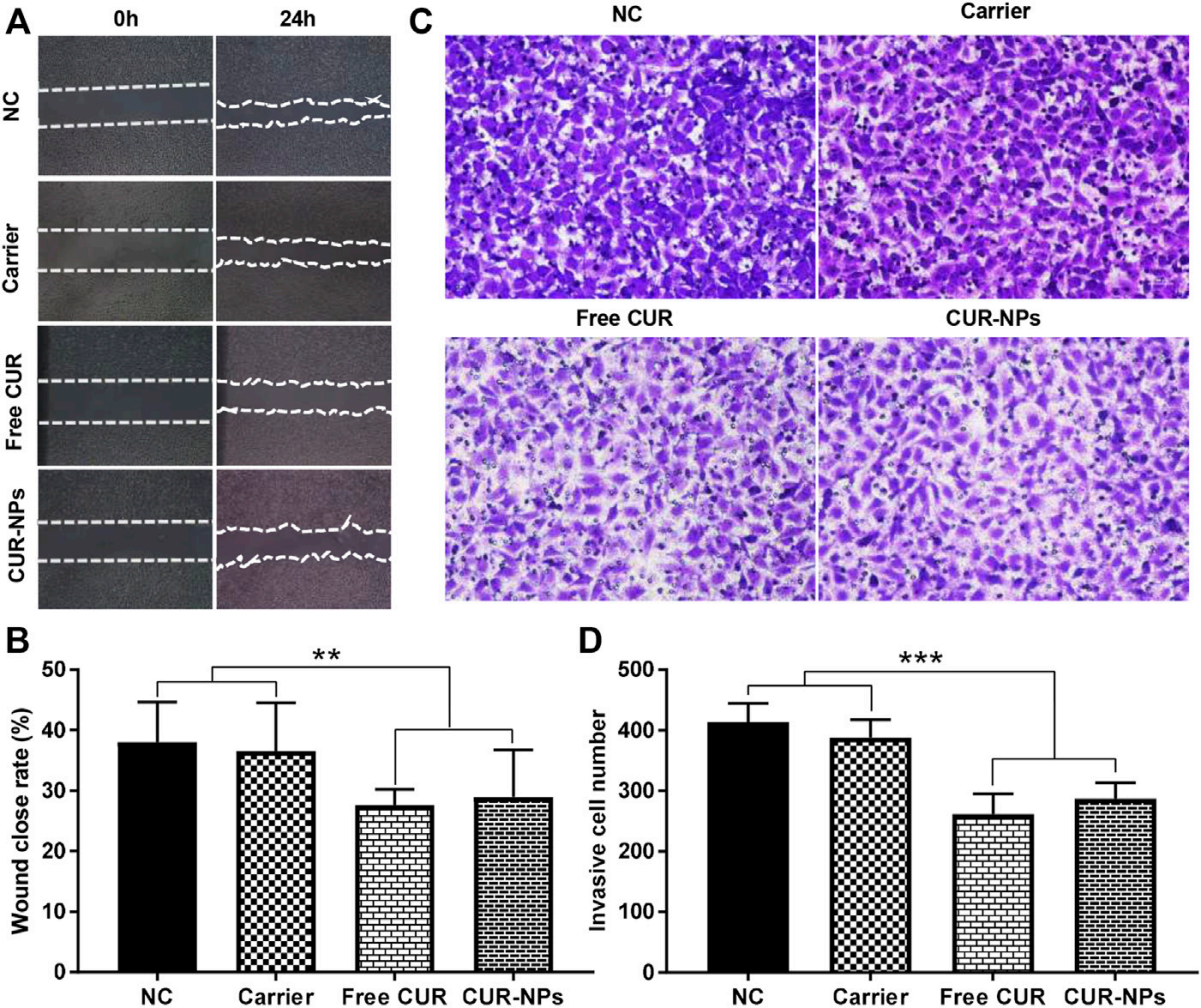
Free CUR and CUR-NPs suppress the migration and invasion of MDA-MB-231 cells. **(A,B)** A wound-healing assay was performed to investigate the migration of MDA-MB-231 cells after being treated with free CUR, CUR-NPs, or carrier. **(C,D)** The invasion abilities of MDA-MB-231 were studied by the transwell assay after cells were treated with free CUR, CUR-NPs, or carrier. NC, treated with negative control; carrier, treated with the carrier of PEG_5k_-b-PLLA_5k_; CUR, treated with free CUR; CUR-NPs, treated with CUR-loaded PEG_5k_-b-PLLA_5k_ nanoparticles. **p < 0.01, ***p < 0.001.

To study the migration abilities of MDA-MB-231 cells after different treatments, a wound-healing assay was performed. As shown in Figures 9A,B, after incubation with free CUR or CUR-NPs, the wound closure rates of MDA-MB-231 cells were 27.60% and 28.97%, which were significantly lower than that of the negative control group (38.02%), indicating that both free CUR and CUR-NPs have capabilities to suppress the migration of MDA-MB-231 cells. To investigate the invasion capability of MDA-MB-231 cells, a transwell invasion assay was used. As depicted in Figures 9C,D, the invasive cell numbers in the lower chamber after the treatment with free CUR or CUR-NPs were 262 and 287, respectively, which were obviously lower than the treatment with the negative control group (414), indicating that both free CUR and CUR-NPs have capabilities to suppress the invasion of MDA-MB-231 cells. Moreover, there were no significant differences in the wound closure rate and the invasive cell number in the lower chamber between the group treated with PEG_5k_-b-PLLA_5k_ and the negative control group, meaning that the ability of CUR-NPs to inhibit the migration and invasion of breast cancer cells mainly attribute to CUR encapsulated in CUR-NPs.

### Downregulation of mRNA expression levels of top 10 genes

**FIGURE 10:**
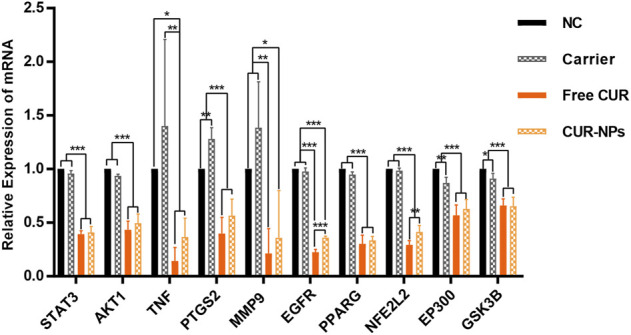
Real-time PCR analysis for the expression of top 10 genes in MDA-MB-231 cells. NC, treated with negative control; carrier, treated with the carrier of PEG_5k_-b-PLLA_5k_; CUR, treated with free CUR; CUR-NPs, treated with CUR-loaded PEG_5k_-b-PLLA_5k_ nanoparticles. *p < 0.05, **p < 0.01, ***p < 0.001.

To investigate the effects of different treatments on the screened top 10 genes, the mRNA expression levels of *STAT3, AKT1, TNF, PTGS2, MMP9, EGFR, PPARG, NFE2L2, EP300,* and *GSK3B* were detected by RT-PCR. After 48 h treatment, the expression levels of the ten genes were apparently inhibited in MDA-MB-231 cells after being treated with both free CUR and CUR-NPs (Figure 10).

### Suppression of the JAK-STAT3 signaling pathway

**FIGURE 11:**
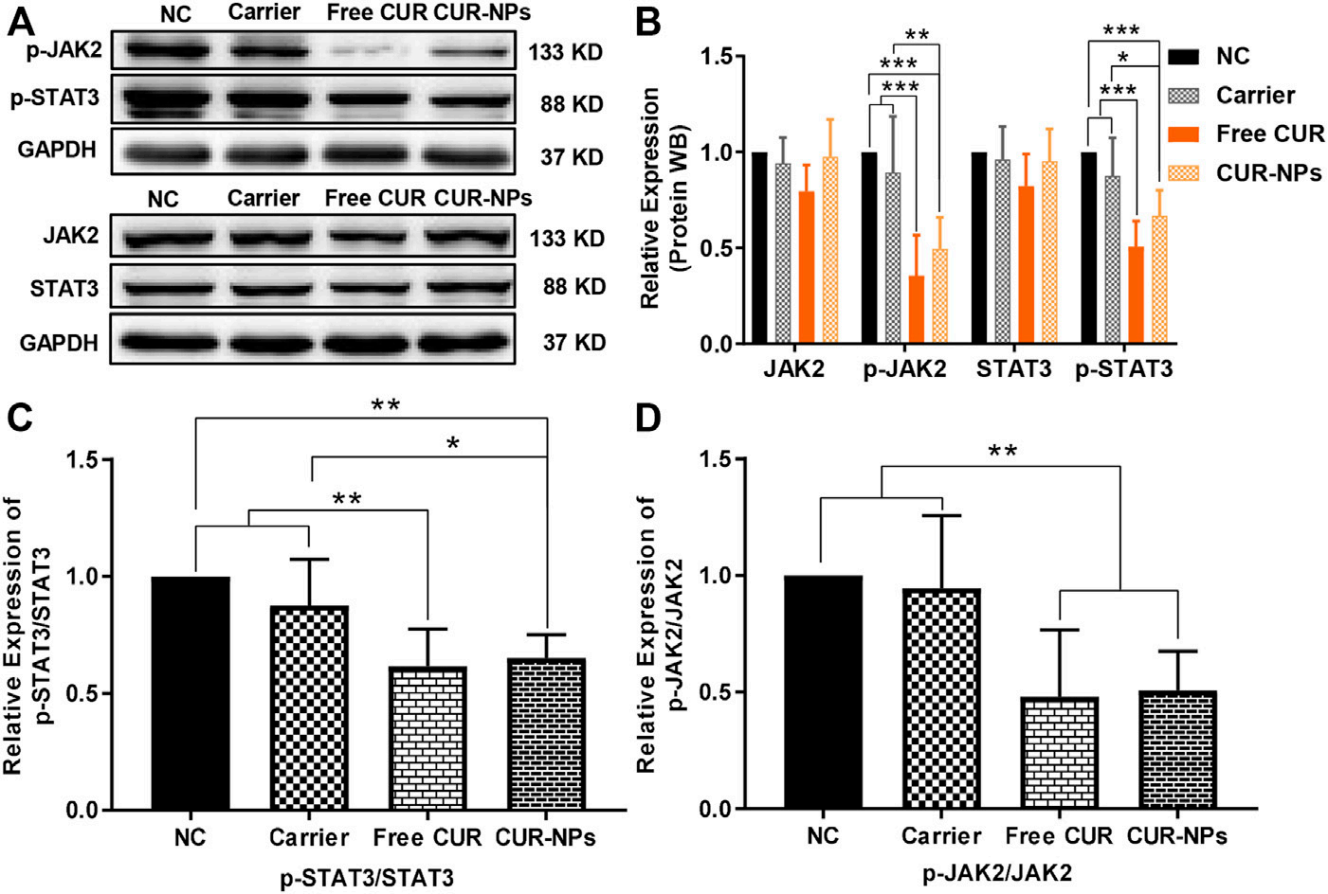
Effects of CUR and CUR-NPs on the expressions of proteins related to the JAK/STAT3 signaling pathway in MDA-MB-231 cells. **(A,B)** The protein expression levels of JAK2, p-JAK2, STAT3, and p-STAT3 were detected by Western blotting. **(C)** The ratio of p-STAT3/STAT3 was relative to the negative control group. **(D)** The ratio of p-JAK2/JAK2 was relative to the negative control group. *p < 0.05, **p < 0.01, ***p < 0.001.

To further verify the suppression of the JAK-STAT3 signaling pathway by free CUR and CUR-NPs, the phosphorylation status of STAT3 (try705) and JAK2 (Tyr1007/1008) residue were determined by immunoblotting. It was found that both the levels of phosphorylated STAT3 and JAK2 were obviously suppressed, however, there were no significant changes in the total levels of STAT3 and JAK2 proteins (Figures 11A,B). The ratios of p-STAT3/STAT3 and p-JAK2/JAK2 were reduced remarkably after being treated with both free CUR and CUR-NPs for 48 h (Figures 11C,D). These results demonstrated that both free CUR and CUR-NPs have the capabilities to suppress the JAK-STAT3 signaling pathway.

## Discussion

TNBC still lacks effective targeted therapy due to the negative expression of human epidermal growth factor receptor 2. Treatments with chemotherapeutic drugs such as taxanes, anthracyclines, and platinum-based agents are the major choice for patients with TNBC. Unfortunately, there are many problems with chemotherapy in the clinic, for example, the chemotherapeutic drugs have serious cytotoxicities and side effects, and patients develop resistance to the chemotherapeutic drugs. Therefore, it is worth developing a new drug with diminished toxicity for the treatment of TNBC. CUR extracted from turmeric exhibits a lot of pharmacological effects such as antioxidant, anti-aging, and anticancer activities. To comprehensively and systematically elucidate the effects and mechanisms of CUR on TNBC, network pharmacology which is a powerful tool to study the complex mechanisms of drug formulations was used in the present study.

40 potential targets of CUR against TNBC were identified by taking the intersection of CUR- and TNBC-related targets. Through analysis of the constructed PPI network, STAT3, AKT1, TNF, PTGS2, MMP9, EGFR, PPARG, NFE2L2, EP300, and GSK3B were identified as the top 10 potential targets of CUR against TNBC. STAT3 is pivotal in regulating the progression, metastasis, and immune evasion of TNBC (Qin et al., 2019). In fact, CUR has been reported to suppress the proliferation, migration, and invasion of cancer cells by suppressing STAT3 activity (Datta et al., 2018; Liang et al., 2021). AKT1 increases the proliferation and survival of cancer cells and AKT inhibitors could control the progression, drug resistance, and immunosuppression of breast cancer (Hinz and Jücker, 2019; Jabbarzadeh Kaboli et al., 2020). TNF often overexpresses in tumors including TNBC and triggers the activation and progression of tumors by enhancing proliferation, transformation, angiogenesis, and metastasis (Yu et al., 2020). PTGS2, also known as COX-2, is overexpressed in TNBC and associated with a poor prognosis (Mosalpuria et al., 2014). CUR displayed an effect on inhibiting the activity of COX-2 (Mortezaee et al., 2019). MMP-9 is a vital component of the metastatic niche during tumorigenesis and can promote the breast cancer cells to colonize the lungs (Owyong et al., 2019). CUR was reported to regulate the metastasis of breast cancer cells *via* inhibiting MMP-9 and MMP-2 (Hassan and Daghestani, 2012). About half of TNBC that overexpress EGFR and CUR could induce cell apoptosis by suppressing the expression of EGFR (Masuda et al., 2012; Sun et al., 2012). In our research, MDA-MB-231 cells were used as a cell model to study the effect of CUR on TNBC. It was demonstrated that CUR could not only suppress the migration, invasion, and proliferation of MDA-MB-231 cells but also induce their apoptosis. From the results of molecular docking and RT-PCR experiment, it could be concluded that CUR may have the capability of binding spontaneously with STAT3, AKT1, TNF, PTGS2, MMP9, EGFR, PPARG, NFE2L2, EP300, and GSK3B and downregulate the genetic expressions of these targets, respectively. To comprehensively and systematically analyze the functions and pharmacological mechanisms of CUR against TNBC, a CUR-targets-pathways network was constructed. According to the constructed network, EGFR tyrosine kinase inhibitor resistance, PI3K-Akt signaling pathway, PD-L1 expression and PD-1 checkpoint pathway in cancer, hemical carcinogenesis-receptor activation, JAK-STAT signaling pathway, and MicroRNAs in cancer were identified as the important pathways of CUR effects on TNBC. Among these signaling pathways, the JAK-STAT signaling pathway is representative and further demonstrated with *in vitro* experiments in our research. Aberrated activation of JAK-STAT signaling contributes to the occurrence, proliferation, invasion, and metastasis of tumors (Khan et al., 2020). Cytokines such as interleukins attach to their receptors and induce the phosphorylation of JAK and STAT, after the phosphorylation, a dimer of STAT is formed, the formed dimer enters the nucleus to attach to DNA and initiates the expression of genes that facilitate the angiogenesis, survival, and proliferation of tumors (Khan et al., 2020). The JAK-STAT signaling could directly or indirectly regulate the NF-κB pathway, interferon-alpha receptor1/2 binds with their receptors to phosphorylate JAK-STAT3 and triggers the activation of the NF-κB pathway *via* PI3K-Akt or TNF receptor-associated factors (Owen et al., 2019). Inhibiting the JAK-STAT signaling pathway can hinder the expression levels of the related target genes that regulate and control the apoptosis, proliferation, and metastasis of cancer. In our research, we demonstrated that CUR can block the phosphorylation of JAK1 and STAT3 of MDA-MB-231 cells, indicating that CUR has the potential capability of suppressing the JAK-STAT signaling pathway of TNBC.

Although CUR exhibits the potential capability of preventing the proliferation, invasion, and metastasis of TNBC, its clinical translation is restricted by the poor solubility and instability in physiological conditions. So, it is urgent to solve these problems of CUR. Nanoscale drug delivery systems such as a nanoparticle, liposome, and nano-emulsion have been reported to have the capabilities of addressing these needs. A nano-emulsion of CUR was reported to improve the solubility, bioavailability, and antioxidant activity of CUR (Mokaberi et al., 2021). Nano-formulated CUR (SinaCurcumin^®^) which exhibited a significantly higher bioavailability for oral consumption was demonstrated to suppress breast cancer cells *via* inhibiting the expression of cyclinD1 (Hosseini et al., 2019). Li et al. (2014) developed a highly water-soluble CUR nano-formulation and demonstrated that the developed CUR nano-formulation could obviously prevent the growth of tumors *in vivo* as compared with free CUR. Lai et al. (2021) constructed a nanoparticle using an amphiphilic hyaluronic acid-CUR conjugate and demonstrated that the constructed nanoparticle could efficiently accumulate in tumors and achieve superior antitumor ability *in vivo*.

Some proteins have the ability to bind drugs and nanoparticles and affect the targeted ability of nanoparticles (Chamani et al., 2005; Abdollahpour et al., 2011; Abdollahpour et al., 2016), and PEG is applied extensively in reducing serum protein adsorption in biological settings and prolonging blood circulation time of nanoparticles, due to its nontoxic, nonimmunogenic, and protein-resistance properties (Cai et al., 2021; von Baeckmann et al., 2021). In our study, CUR-NPs were prepared by using PEG_5k_-b-PPLA_5k_ as the carrier to encapsulate CUR, and then the *in vitro* experiments were used to further verify the effect and mechanism of CUR in the formulation of nanoparticles. PEG_5k_-b-PLLA_5k_ is an amphiphilic block copolymer, which can form core-shell nanoparticles with PEG as the shell (Rabanel et al., 2015). So, it is assumed that a very weak interaction will occur between proteins and the nanoparticles prepared with PEG_5k_-b-PLLA_5k_. Moreover, it was found from western blot results of our research that PEG_5k_-b-PLLA_5k_ has little effect on the expression levels of phosphorylated STAT3 and JAK2, which also indirectly proves that the nanoparticles prepared with PEG_5k_-b-PLLA_5k_ have a low possibility of interacting with targeted proteins. Overall, our results demonstrated that CUR-NPs could obviously enhance the water solubility of CUR, but have little effect on the mechanism of CUR against TNBC.

## Conclusion

Taken together, using the combination of network pharmacology, molecular docking, and *in vitro* bio-experiment validation, we demonstrate that CUR and CUR-NPs have the capabilities to suppress the migration and invasion, restrain the proliferation, and induce the apoptosis of TNBC cells, mainly by downregulating the genetic expression of *STAT3, AKT1, TNF, PTGS2, MMP9, EGFR, PPARG, NFE2L2, EP300,* and *GSK3B* and inhibiting JAK-STAT signaling pathway.

## Data Availability

The original contributions presented in the study are included in the article/Supplementary Material, further inquiries can be directed to the corresponding authors.
