# Supplementary material for: Curcumin and its nano-formulations: Defining triple-negative breast cancer targets through network pharmacology, molecular docking, and experimental verification
Source: Front Pharmacol. 2022 Aug 8;13:920514. doi: 10.3389/fphar.2022.920514 (PMC9393234; doi:10.3389/fphar.2022.920514)
Supplement: Supplementary file 4 [file Table4.DOCX]

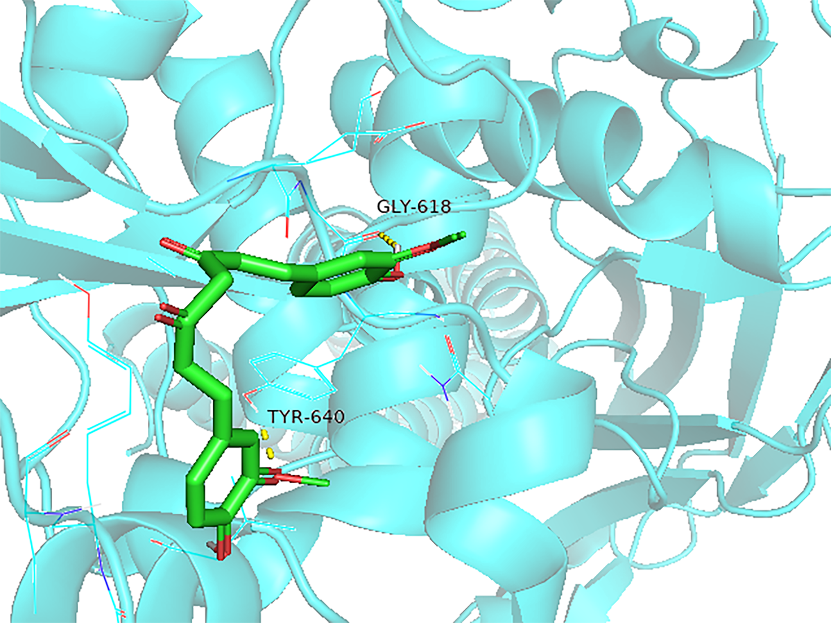


#### Supplementary Figure S1 Molecular docking result of CUR with STAT3(PDB ID:6NJS). CUR and two protein residues (GLY-618 and TYR-640) in STAT3 bonded to each other through hydrogen bonds.


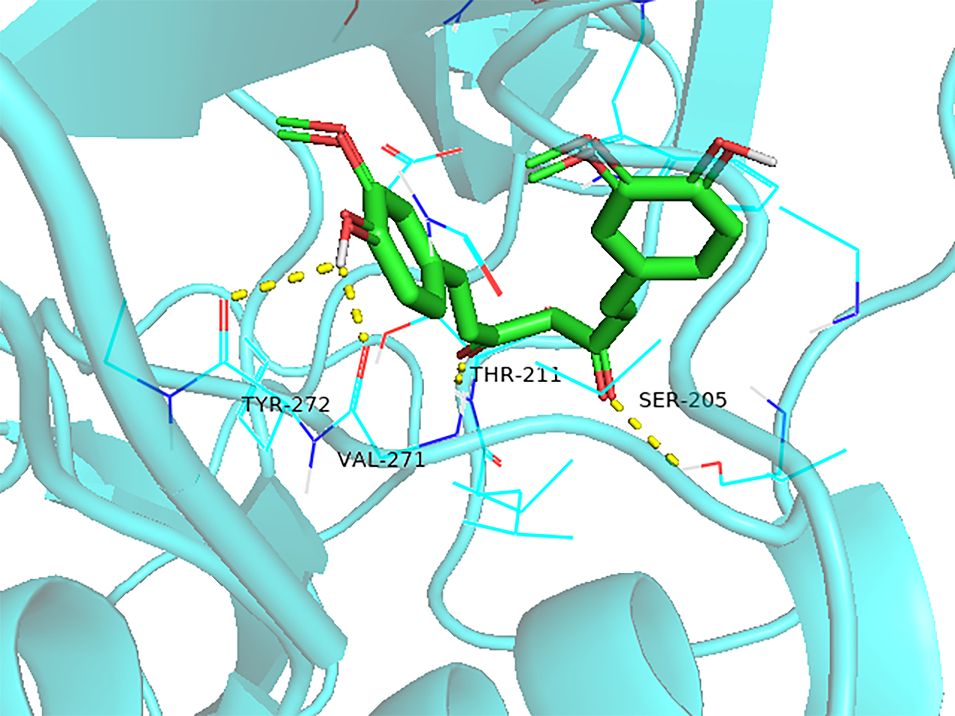


#### Supplementary Figure S2 Molecular docking result of CUR with AKT1(PDB ID:4EJN). CUR and four protein residues (TYR-272, VAL-271, THR-211 and SER-205) in AKT1 bonded to each other through hydrogen bonds.

**
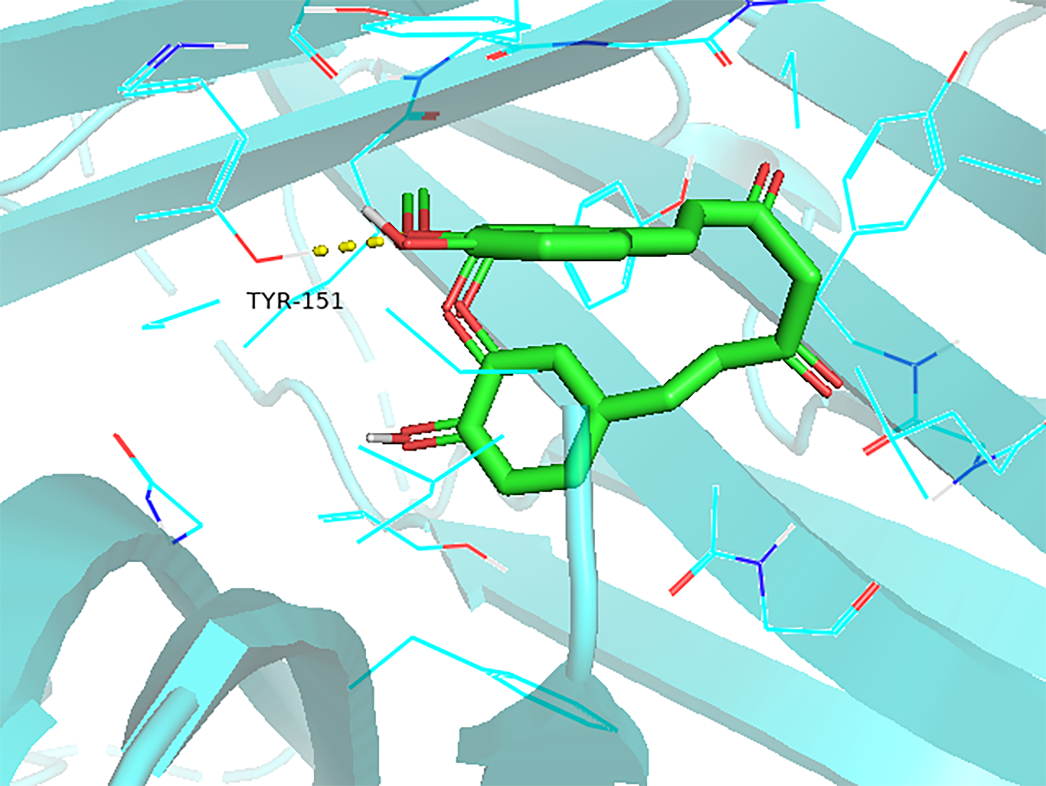
**

#### Supplementary Figure S3 Molecular docking result of CUR with TNF (PDB ID:6OOZ). CUR and one protein residues (TYR-151) in TNF bonded to each other through hydrogen bonds.

**
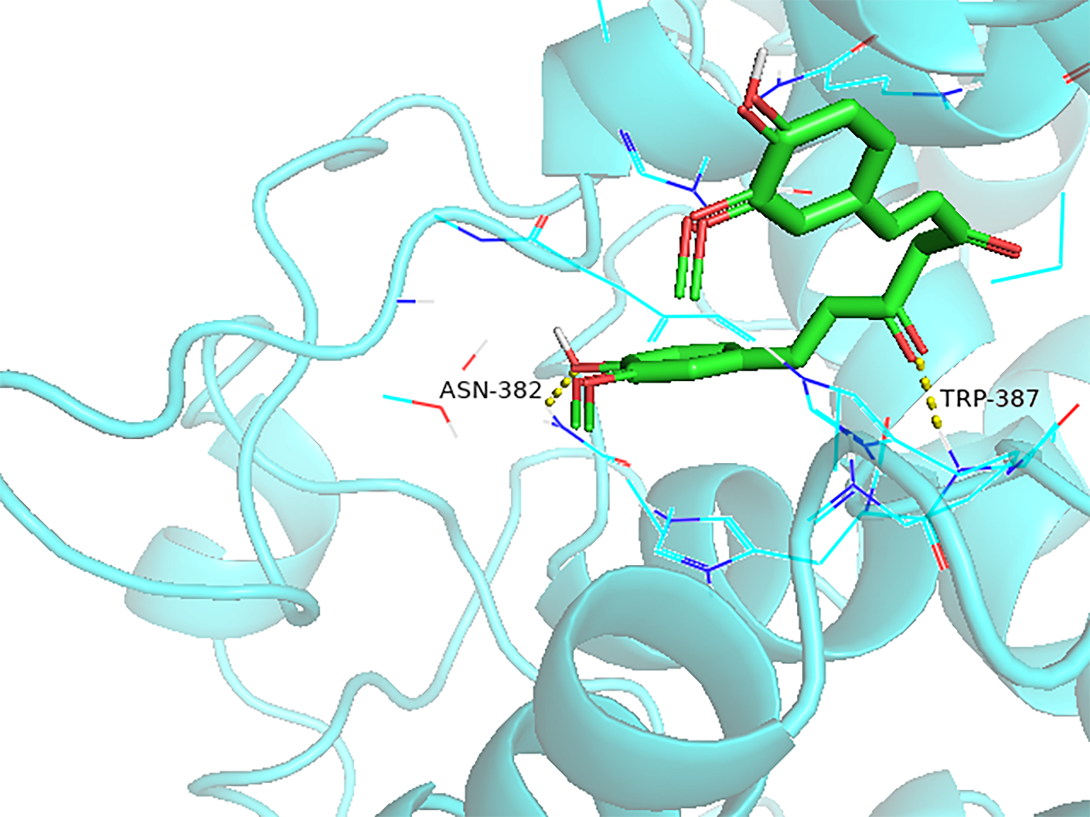
**

#### Supplementary Figure S4 Molecular docking result of CUR with PTGS2 (PDB ID:5F1A). CUR and two protein residues (ASN-382 and TRP-387) in PTGS2 bonded to each other through hydrogen bonds.


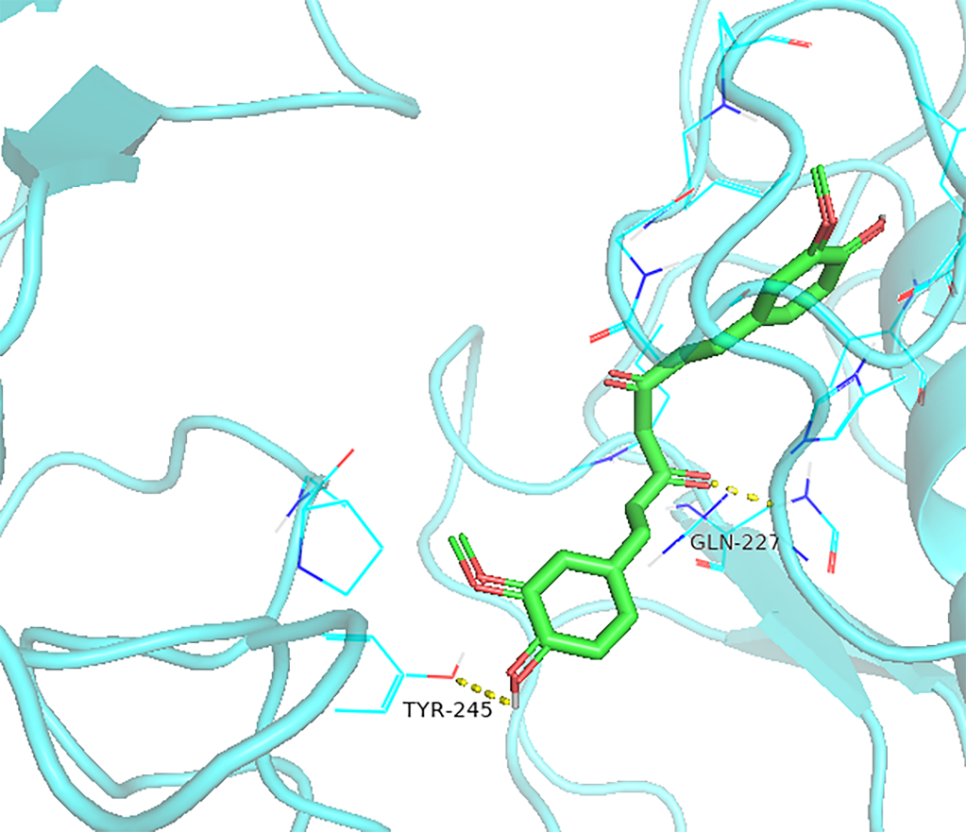


#### Supplementary Figure S5 Molecular docking result of CUR with MMP9 (PDB ID:4HMA). CUR and two protein residues (TYR-245 and GLN-227) in MMP9 bonded to each other through hydrogen bonds.


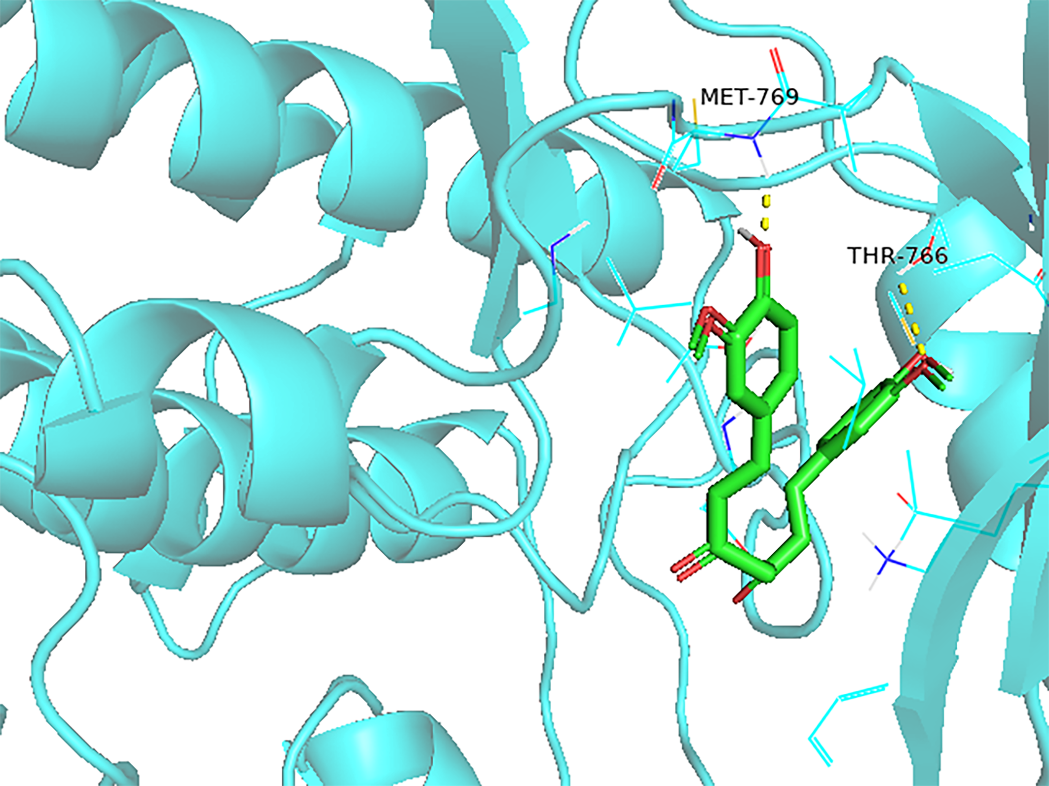


#### Supplementary Figure S6 Molecular docking result of CUR with EGFR (PDB ID:1M17). CUR and two protein residues (THR-766 and MET-769) in EGFR bonded to each other through hydrogen bonds.


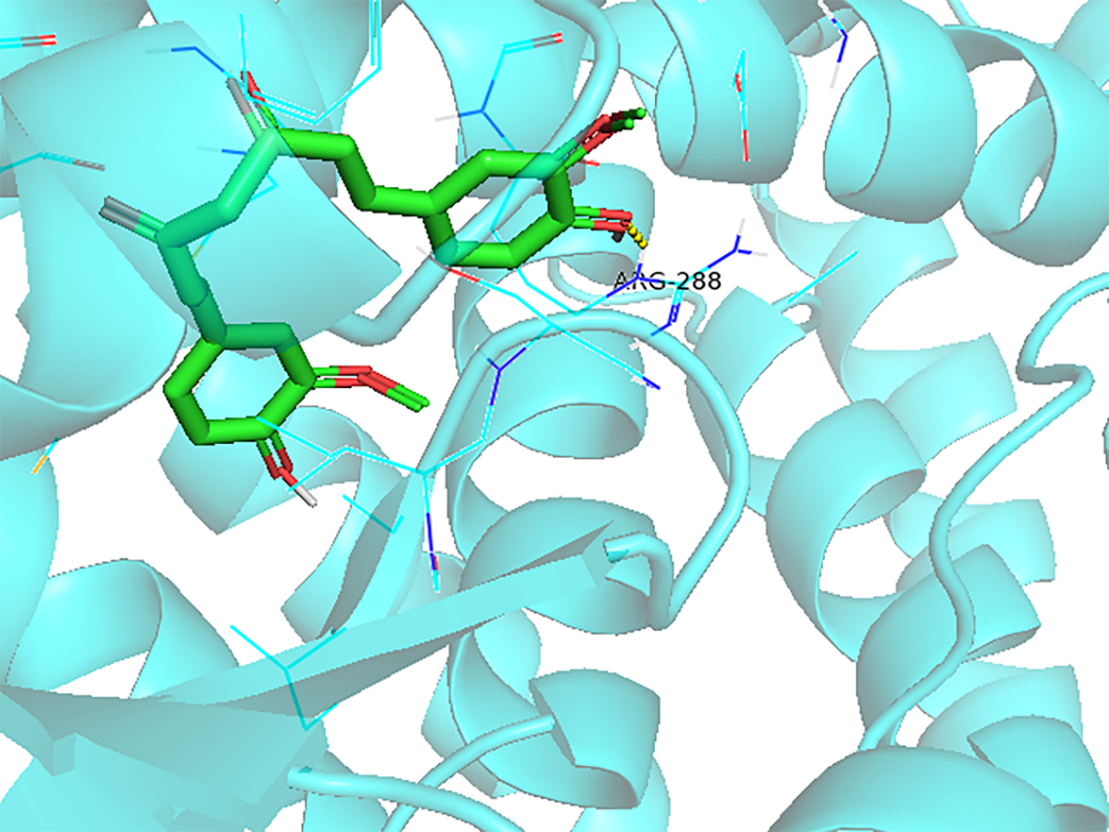


#### Supplementary S7 Molecular docking result of CUR with PPARG (PDB ID:5UGM). CUR and one protein residues (ARG-288) in PPARG bonded to each other through hydrogen bonds.


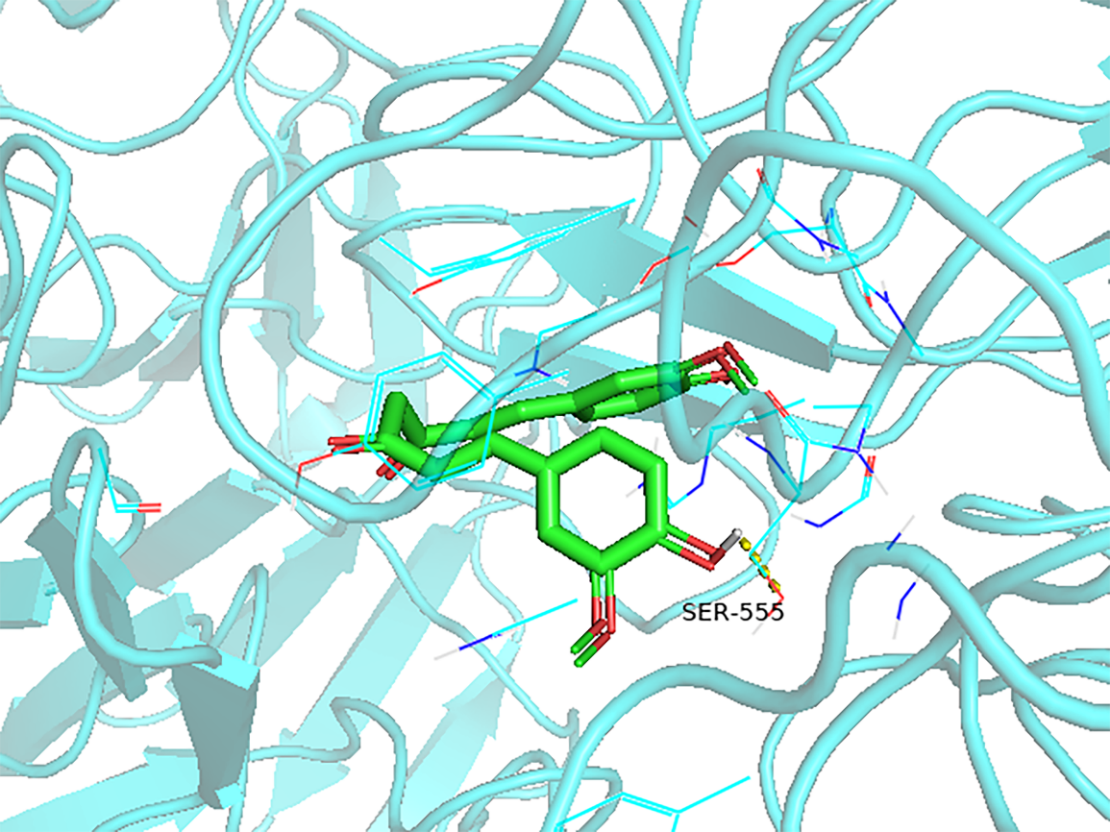


#### Supplementary Figure S8 Molecular docking result of CUR with NFE2L2 (PDB ID:3ZGC). CUR and one protein residues (SER-555) in NFE2L2 bonded to each other through hydrogen bonds.


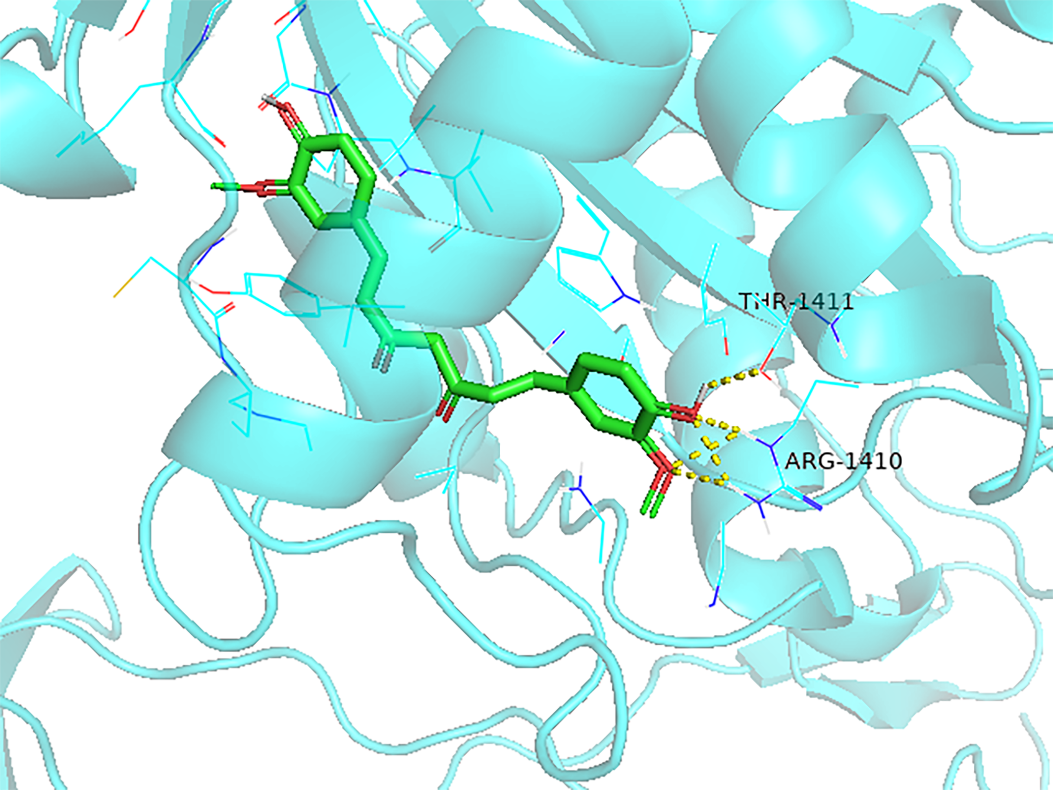


#### Supplementary Figure S9 Molecular docking result of CUR with EP300 (PDB ID:5LKT). CUR and two protein residues (THR-1411 and ARG-1410) in EP300 bonded to each other through hydrogen bonds.


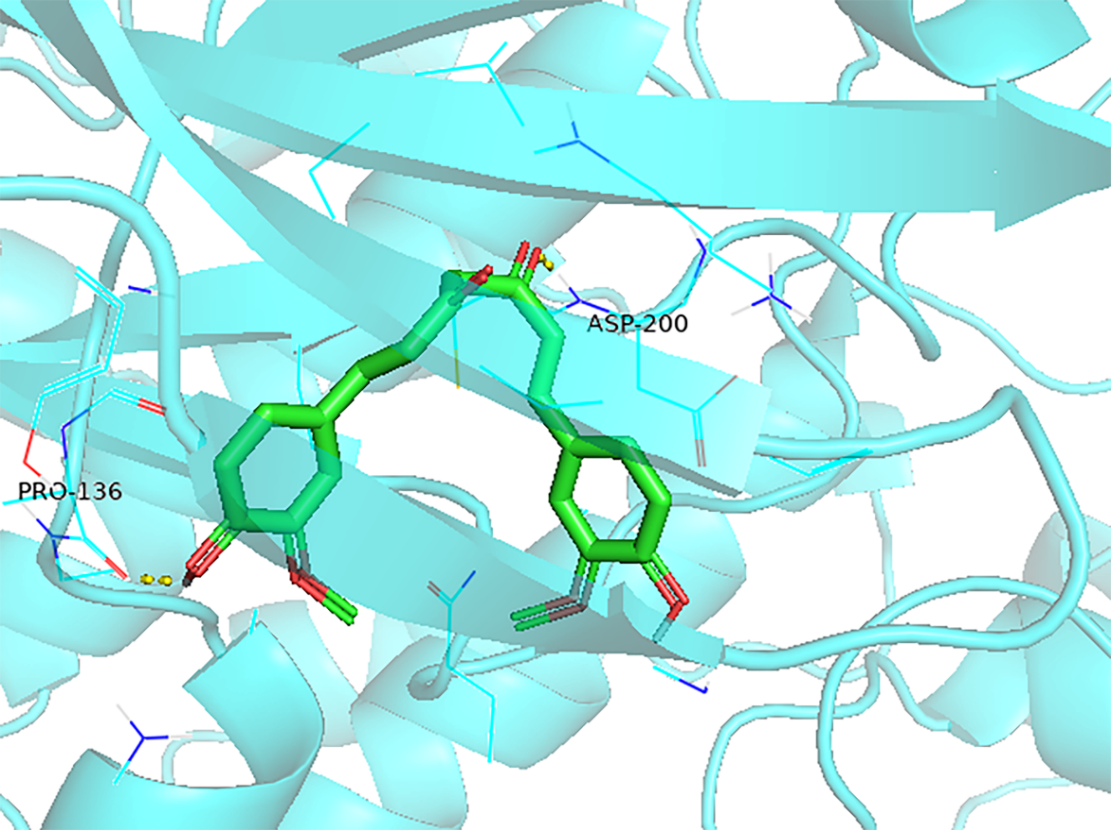


#### Supplementary Figure S10 Molecular docking result of CUR with GSK3B (PDB ID:4J71). CUR and two protein residues (PRO-136 and ASP-200) in GSK3B bonded to each other through hydrogen bonds.


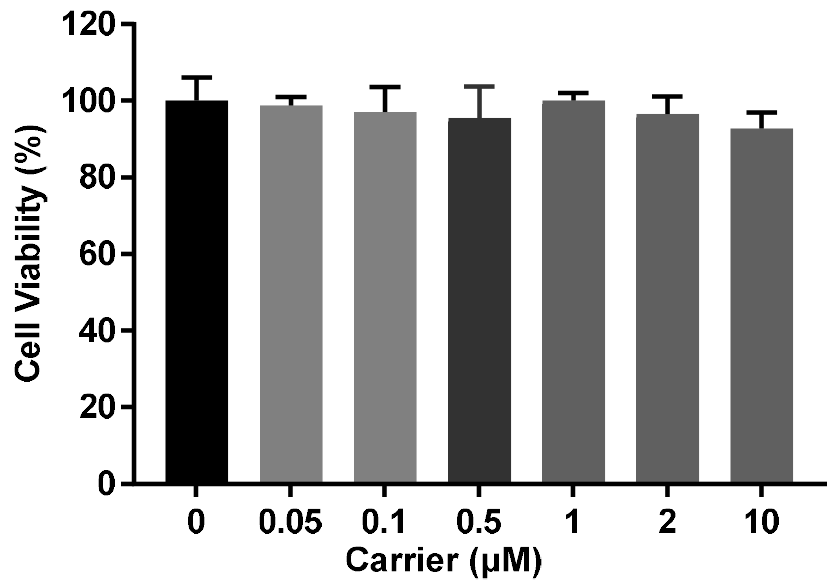


#### Supplementary Figure S11. The cytotoxicity of the carrier (PEG_5K_-b-PLLA_5K_) on MDA-MB-231 cells
